# Beckwith-Wiedemann syndrome and twinning: case report and brief review of literature

**DOI:** 10.1186/s13052-023-01530-8

**Published:** 2023-09-25

**Authors:** Pierandrea Elefante, Beatrice Spedicati, Flavio Faletra, Laura Pignata, Flavia Cerrato, Andrea Riccio, Egidio Barbi, Luigi Memo, Laura Travan

**Affiliations:** 1 Department of Medicine, Surgery, and Health Sciences, University of Trieste, Via dell’Istria 65/1, Trieste, 34137 Italy; 2 Medical Genetics, Institute for Maternal and Child Health, IRCCS Burlo Garofolo, Trieste, Italy; 3 Department of Environmental Biological and Pharmaceutical Sciences and Technologies (DiSTABiF), Università degli Studi della Campania “Luigi Vanvitelli”, Caserta, Italy; 4 Institute of Genetics and Biophysics (IGB) “Adriano Buzzati-Traverso”, Consiglio Nazionale delle Ricerche (CNR), Naples, Italy; 5 Institute for Maternal and Child Health, IRCCS Burlo Garofolo, Trieste, Italy; 6 Clinical Genetics, Department of Pediatrics, Ospedale San Bortolo, Vicenza, Italy; 7 Neonatal Intensive Care Unit, Institute for Maternal and Child Health, IRCCS Burlo Garofolo, Trieste, Italy

**Keywords:** Beckwith-wiedemann syndrome, Twinning, Imprinting disorders, Case report

## Abstract

**Background:**

Beckwith-Wiedemann syndrome (BWS, OMIM #130,650) is a pediatric overgrowth disorder involving a predisposition to tumor development. Although the clinical management of affected patients is well established, it is less clear how to handle with the cases of siblings of affected patients, since the prevalence of the condition in twins (1:1000) is ten times higher than in singletones (1:10000).

**Case presentation:**

We report the case of a premature twin patient who during her follow-up develops a clinical phenotype compatible with BWS, genetically confirmed in blood. However, the methylation alteration characteristic of the condition was also found in the almost phenotypically normal sibling, making it challening her management.

**Conclusion:**

Through our case report we highlight how the diagnosis of BWS can be made without any prenatal suspicion and we propose a review of the literature on how to manage siblings of affected patients in twinning situation.

## Background

Beckwith-Wiedemann syndrome (BWS) is the most common genetic overgrowth disorder, with an estimated prevalence of 1:10000 live births [1]. The association between the syndrome and twins, where the incidence is significantly increased [2] up to about 10 times compared to the one previously reported, has been known for a long time [3] and how to manage siblings affected by the condition has been a long-standing question. However definitive guidelines are lacking, making it challenging to handle with the sibling of a newly-diagnosed patient.

## Case presentation

A couple of female monochorionic-biamniotic twins was born preterm at 28 ^6/7^ weeks of gestational age by a vaginal delivery due to unstoppable labour. No infectious nor familiar risk factors were reported, and pregnancy developed spontaneously. Antepartum betamethasone treatment was completed. Newborn adaptation was regular, with an Apgar score of 8–10 at 1 and 5 min respectively for the first twin (FT), and 6–9 for the second twin (ST).

The twins had an appropriate weight for gestational age (AGA), albeit at medium-high percentiles (72°pc and 85°pc according to Bertino [4], respectively). Upon clinical examination, FT presented a hemangioma precursor of the right hand and wrist while ST presented a mild reducible umbilical hernia; both of them presented camptodactyly. Perinatal clinical course was unremarkable for each twin, except for a mild precocious hypoglycemia requiring intravenous correction in ST, with prompt normalization of glucose values.

**Fig. 1:**
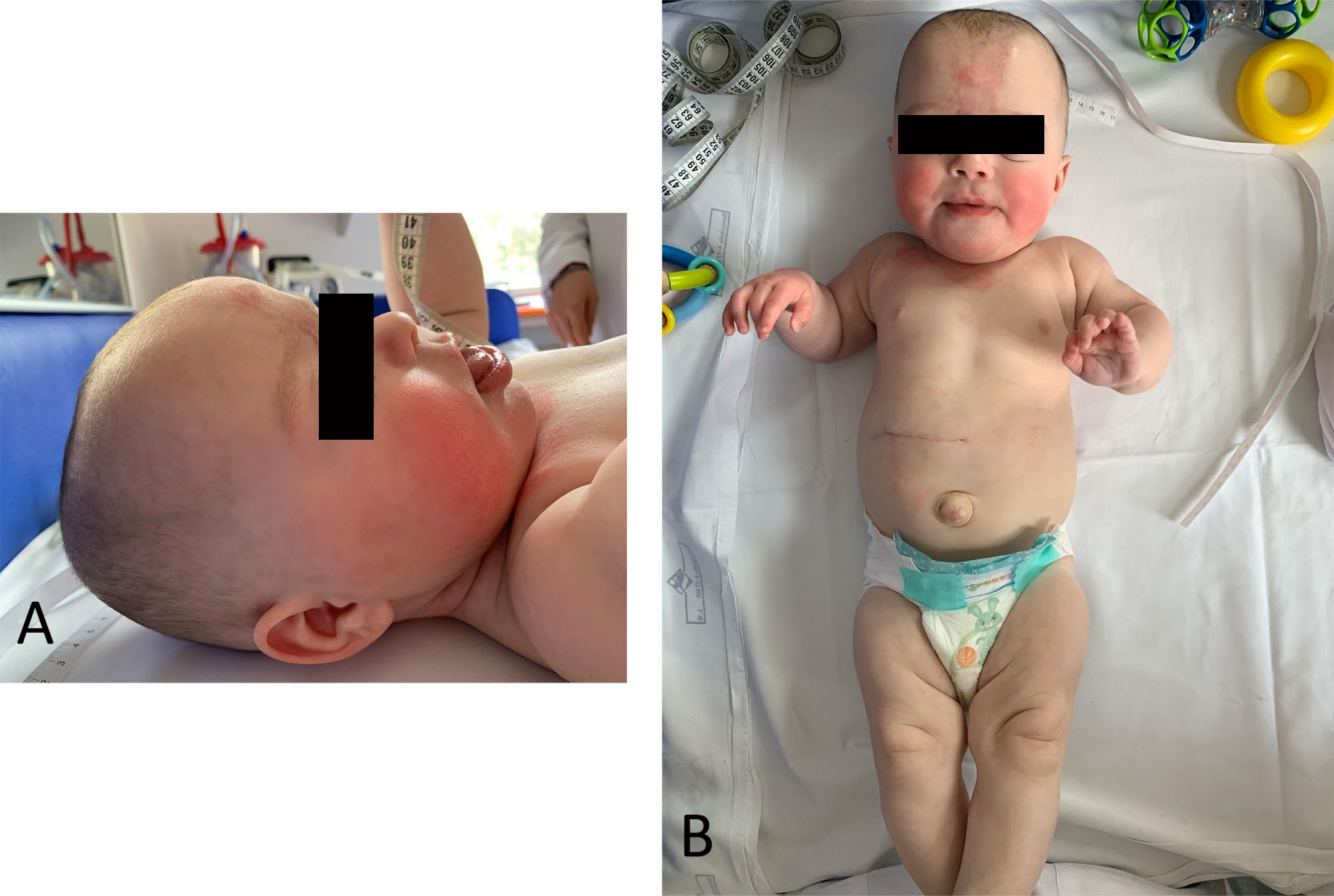
(**A**) ST Profile view: note the macroglossia and the ear lobe creases, two typical signs of BWS. While macroglossia is usually evident, ear creases might be missed if not specifically searched. (**B**) ST: Wide glabellar nevus simplex, reducible umbilical hernia and a scar in right hypocondrium due to the surgical excision of the adrenal neuroblastoma

The twins were discharged at 37 ^2/7^ weeks of postconceptional age in good general conditions. However, during the first follow-up evaluation at 3 months of chronological age, the clinical presentation of the two infants split: while FT growth seemed to proceed regularly, ST presented with a significant weight increase (reaching 97°pc), a wide glabellar nevus simplex, and moderate macroglossia (Fig. 1A). The umbelical hernia had grown larger, though still being reducible (Fig. 1B). No hemihypertrophy was perceptible. According to *BWSp scoring system* [5], a clinical diagnosis of classic BWS could be established. A blood sample to perform a Methylation-specific multiple ligation-dependent probe amplification (MS-MLPA) analysis of the BWS critical region (11p15.5-p15.4 locus) was drawn from each twin.

While awaiting for the genetic test result, during the first ST surveillance ultrasound of the abdomen, an adrenal cystic mass was found: the following meta-iodobenzylguanidine (MIBG) scintigraphy confirmed the first suspect of a cystic neuroblastoma, which was successfully surgically removed. Meanwhile, genetic investigations identified a loss of methylation (LOM) of the *KCNQ1OT1*:TSS DMR (or Imprinting Centre 2, IC2) thus confirming the clinical diagnosis: the methylation alteration was detectable in both twins, also in the one who did not express a clear BWS phenotype. Methylation analysis was then performed on DNA extracted from buccal mucosa by pyrosequencing. In this case, IC2 LOM was confirmed in ST, while the methylation level of FT was comparable to that of normal controls.

## Discussion and conclusions

Twinning, and in particular female-female twinning [6], is known to be correlated with BWS and, according to some authors, the presence of DNA methylation defect associated to the syndrome could represent the initial trigger for the twinning event [7]. The broad spectrum of clinical presentations in monozygotic twins has been justified by Cohen et al. [8]. as depending on the different timing of both epigenetic error and twinning event (the so-called “diffused mosaicism theory”). Moreover, other Authors, as a corollary mechanism to diffused epigenetic mosaicism, have developed the “epigenetic burden theory”: according to this theory, it may exist a tissue-dependent threshold for the number of abnormal cells required to develop a pathological phenotype [9].

**Fig. 2:**
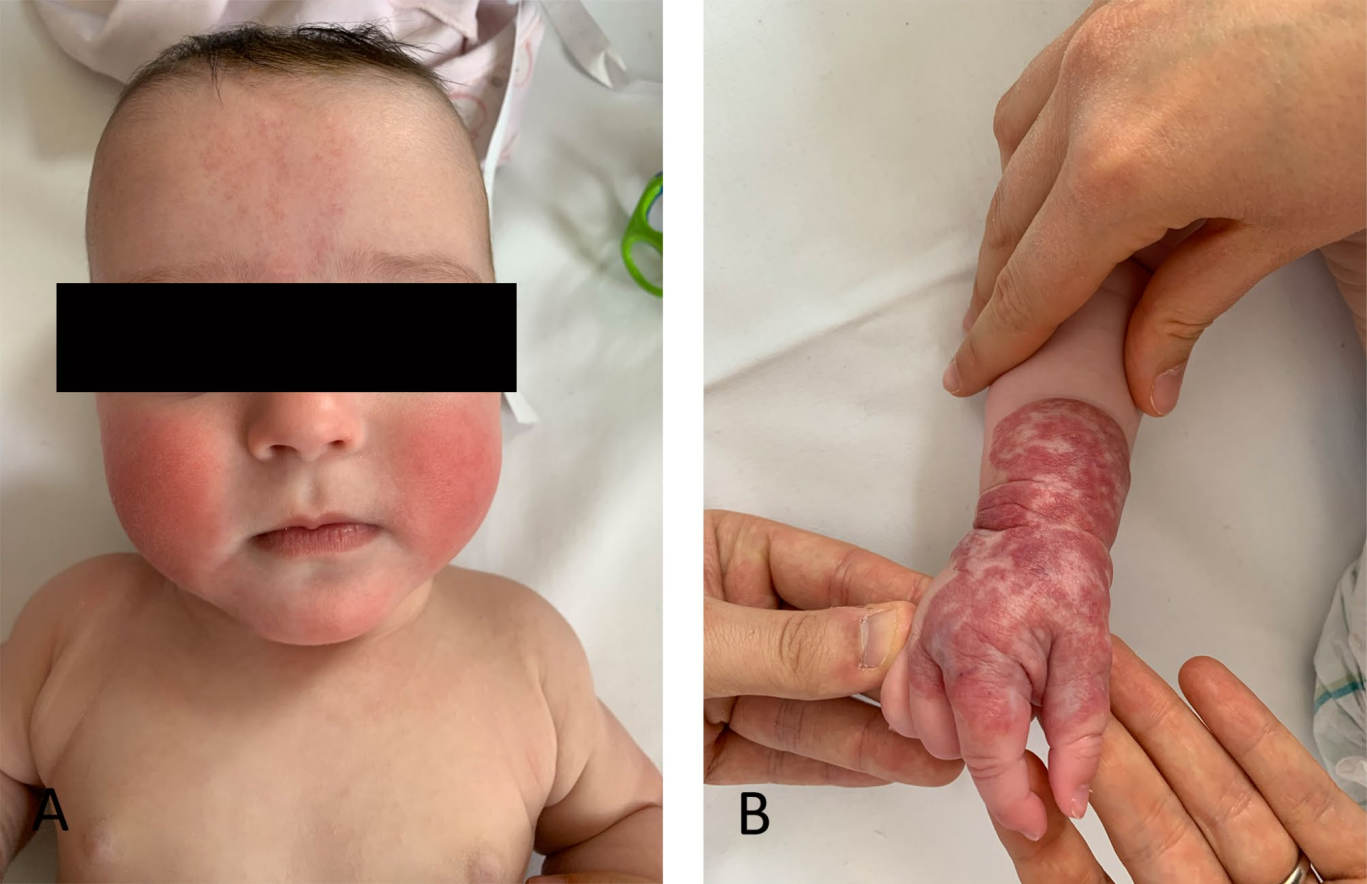
(**A**) Less affected sibling (FT) presenting normal facies with no macroglossia but a nuanced glabellar nevus simplex. (**B**) FT: Hemangioma of the right hand and wrist. Hemangiomas are known to be clinical features associated with BWS [6] although not being part of the clinical diagnostic score

These considerations make it often tricky to clinically manage the less phenotypically affected sibling: in our case, one twin (FT) only presented a mild glabellar nevus simplex and a hemangioma of the right wrist and hand (Fig. 2A and B), which theoretically awarded her a *BWSp Score* of just one [5]. However, because of the remarkable medical history of her sister and according with parental will, we provided to initiate the tumor surveillance protocol for her as well, consistent with what proposed by Cohen [8]. Until now, the first three-years oncological follow-up has always resulted negative.

Our case demonstrates how the management of twins affected by BWS is sharply different from that of singletons. We suggest, when approaching to discordant twins, to start immediately the study of the methylation pattern even on tissues other than blood, for example buccal swab: in fact, it seems well established that hypomethylation can be carried in blood cells without a pathological phenotype, probably due to placental anastomosis leading to shared blood precursors carrying the epigenetic defects [10]. Vice versa, Sun et al. described the case of a couple of dichorionic diamniotic monozygotic twins with BWS who had identical *BWSp scores* but nonidentical epigenetic alteration on peripheral blood leukocytes, making it evident as well that tissue-specific analysis should be considered the gold standard for the detection of altered methylation [11].

Furthermore, whether to insert children with IC2 LOM into a strict tumor surveillance is a debated issue: the 2018 International Consensus written by Brioude et al. [5] proposes no routine ultrasound scan surveillance for these patients, given the mildly increased overall tumour risk (2.6%, versus 28.1% affecting those with IC1 Gain of Metilation (GOM)). In our case, we began the ultrasound scan surveillance since genetic tests were still under investigation and due to the low invasiveness of the exam.

Lastly, our case is significative because it demonstrates how typical features (10% regarding macroglossia, 50% for macrosomia) of the syndrome may be absent at birth and develop postnatally, as already described in literature [12]. Obviously, in our case prematurity may have been an additional misleading factor in highlighting the onset of symptoms.

## Data Availability

Not applicable.

## List of abbrevations

BWS: Beckwith-Wiedemann syndrome
FT: First twin
MIBG: Meta-iodobenzylguanidine
MS-MLPA: Multiple ligation-dependent probe amplification
ST: Second twin

## References

[1] Mussa A, Russo S, De Crescenzo A, Chiesa N, Molinatto C, Selicorni A (2013). Preval Beckwith-Wiedemann Syndrome North West Italy Am J Med Genet A.

[2] Weksberg R, Shuman C, Caluseriu O, Smith AC, Fei YL, Nishikawa J (2002). Discordant KCNQ1OT1 imprinting in sets of monozygotic twins discordant for Beckwith-Wiedemann syndrome. Hum Mol Genet.

[3] Franceschini P, Guala A, Vardeu MP, Franceschini D (1993). Monozygotic twinning and Wiedemann-Beckwith syndrome. Am J Med Genet.

[4] Bertino E, Spada E, Occhi L, Coscia A, Giuliani F, Gagliardi L (2010). Neonatal anthropometric charts: the italian neonatal study compared with other european studies. JPGN.

[5] Brioude F, Kalish JM, Mussa A, Foster AC, Bliek J, Ferrero GB (2018). Expert consensus document: clinical and molecular diagnosis, screening and management of Beckwith-Wiedemann syndrome: an international consensus statement. Nat Rev Endocrinol.

[6] Bliek J, Verde G, Callaway J, Maas SM, De Crescenzo A, Sparago A (2009). Hypomethylation at multiple maternally methylated imprinted regions including PLAGL1 and GNAS loci in Beckwith-Wiedemann syndrome. Eur J Hum Genet.

[7] Bestor TH (2003). Imprinting errors and developmental asymmetry. Philosophical Trans Royal Soc Lond Ser B: Biol Sci.

[8] Cohen JL, Duffy KA, Sajorda BJ, Hathaway ER, Gonzalez-Gandolfi CX, Richards-Yutz J (2019). Diagnosis and management of the phenotypic spectrum of twins with Beckwith-Wiedemann syndrome. Am J Med Genet A.

[9] Duffy KA, Hathaway ER, Klein SD, Ganguly A, Kalish JM (2021). Epigenetic mosaicism and cell burden in Beckwith-Wiedemann syndrome due to loss of methylation at imprinting control region 2. Cold Spring Harb Mol Case Stud.

[10] Fontana L, Bedeschi MF, Cagnoli GA, Costanza J, Persico N, Gangi S (2020). Epi)genetic profiling of extraembryonic and postnatal tissues from female monozygotic twins discordant for Beckwith-Wiedemann syndrome. Mol Genet Genomic Med.

[11] Sun F, Hara S, Tomita C, Tanoue Y, Yatsuki H, Higashimoto K (2021). Phenotypically concordant but epigenetically discordant monozygotic dichorionic diamniotic twins with Beckwith-Wiedemann syndrome. Am J Med Genet A.

[12] Shuman C, Beckwith JB, Weksberg R, Beckwith-Wiedemann S. 2000 Mar 3 [Updated 2016 Aug 11]. In: Adam MP, Ardinger HH, Pagon RA, editors. GeneReviews®

